# Revealing Temperature‐Driven Interfacial Reactions of Succinonitrile in PEO‒LiTFSI Electrolytes and Suppressing Them via Dual PEDOT‐Based Interfacial Layers

**DOI:** 10.1002/advs.78096

**Published:** 2026-10-08

**Authors:** Baskar Selvaraj, Hailemichael Ayalew, Yu‐Yao Tsai, Ly Tho Xuan, Jia‐Wei She, Hao‐Wen Liu, Ming‐Chou Chen, Yu‐Sheng Hsiao, Nae‐Lih Wu, Hsiao‐hua Yu

**Affiliations:** ^1^ Smart Organic Materials Laboratory Institute of Chemistry, Academia Sinica Taipei Taiwan; ^2^ Department of Materials Science and Engineering National Taiwan University of Science and Technology Taipei Taiwan; ^3^ Department of Chemical Engineering National Taiwan University Taipei Taiwan; ^4^ Department of Chemistry National Central University Taoyuan Taiwan

**Keywords:** lithium metal batteries, PEDOT‐based interfacial layers, PEO solid polymer electrolytes, succinonitrile (SN), temperature‐driven interfacial reactions, wide‐temperature operation

## Abstract

Achieving a homogeneous ion‐conducting network with sufficient mechanical strength and interfacial stability in Succinonitrile (SN)‐polyethylene oxide (PEO)‐based solid polymer electrolytes remains challenging. In particular, the temperature‐dependent interfacial behavior of SN in PEO‒lithium bis(trifluoromethanesulfonyl)imide (LiTFSI) electrolytes is largely unexplored. Here, we reveal that SN undergoes temperature‐driven interfacial reactions at both lithium metal and cathodes, in addition to reactions involving PEO and LiTFSI. Notably, cathode‐side reactions contribute more to interfacial degradation and capacity fading than the widely studied lithium‐SN interfacial reactions. To address these challenges, a dual‐sided protection strategy using PEDOT‐based interfacial layers is developed. A PEO‒SN‒LiTFSI solid‐polymer electrolyte with high SN content and moderate salt concentration is reinforced with a poly(vinylidene fluoride‐co‐hexafluoropropylene) nanofiber network to maintain mechanical integrity and continuous ion‐conducting pathways. On the anode side, a PEDOT‐based nanofibrous mat coated with a thin gel layer suppresses the transport of reactive electrolyte species toward the lithium interface. On the cathode side, sulfonate‐functionalized PEDOT forms a conformal coating that suppresses interfacial reactions, including those associated with SN. This integrated design enables stable cycling for over 500 cycles and retains >70% capacity at 50°C, demonstrating an effective strategy to suppress temperature‐driven interfacial reactions and achieve stable wide‐temperature (10°C–50°C) solid‐state battery operation.

## Graphical abstract

Temperature‐driven interfacial reactions of succinonitrile in PEO‐LiTFSI electrolyte are revealed at both lithium and cathode interfaces, identifying cathode degradation as the dominant failure pathway. Dual PEDOT‐based interfacial protection suppresses these reactions, stabilizes both electrode interfaces, and enables durable wide‐temperature solid‐state batteries with long‐term cycling stability.

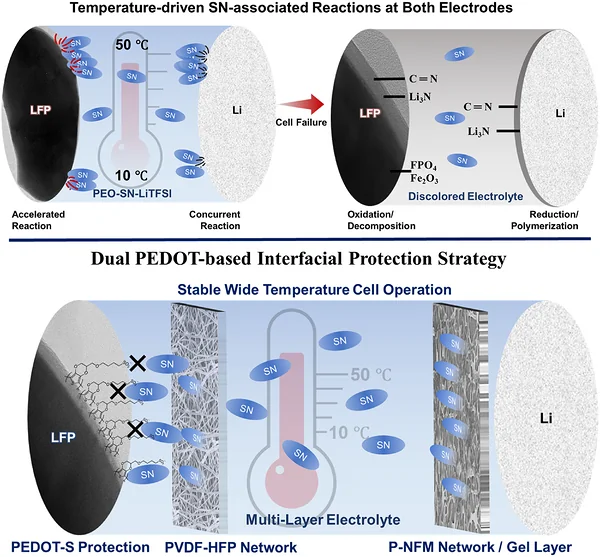

## 1 Introduction

Non‐ionic succinonitrile (SN) has occupied a unique place in the development of solid‐state electrolytes, primarily due to its plastic crystalline phase over a wide temperature range (approximately −40°C to 60°C) and its high polarity, which allows it to dissolve lithium salts [1, 2, 3, 4]. Doping of lithium bis(trifluoromethanesulfonyl)imide (LiTFSI) salt into the defective trans‐isomeric SN matrix has yielded high ionic conductivity, ranging from 10^−4^ to 10^−3^ S cm^−1^ at room temperature (RT), depending on the doping level [5]. An increase in salt concentration enhances ionic conductivity but lowers the melting point of SN, transforming the solid electrolyte into a highly viscous liquid [6]. This viscous form can neither be used directly between electrodes nor with separators due to its lack of mechanical integrity and poor wettability [7]. In addition to this mechanical limitation, lithium‐catalyzed chemical polymerization of nitrile groups further restricts the use of SN‒LiTFSI electrolytes in high‐capacity lithium metal batteries [4]. Therefore, overcoming both mechanical and chemical instabilities is crucial for enabling the practical application of the SN‒LiTFSI system.

Solid‐polymer electrolyte systems are considered among the most promising solutions to address the safety problems associated with conventional liquid electrolytes [4, 8, 9]. However, their practical application remains limited by low ionic conductivity at room temperature, primarily due to the high crystallinity of most polymers. Liquid‐phase plasticizers are therefore incorporated to enhance ionic conductivity, giving rise to gel polymer electrolytes. However, the incorporation of liquid components can retain some of the safety concerns associated with liquid electrolytes and introduce undesired interfacial reactions, while compromising the electrochemical and thermal stability and mechanical strength of the electrolyte [8, 10]. Incorporation of SN‒LiTFSI electrolyte into polymer matrices such as poly(vinylidene fluoride‐co‐hexafluoropropylene) (PVDF‐HFP) and PEO offers a promising alternative to volatile organic liquid components for enhancing ionic conductivity, while exploiting the complementary characteristics of the polymer matrix and SN‐based electrolyte to offset their respective inherent limitations [11, 12, 13, 14, 15, 16, 17, 18, 19]. The interactions of SN‒LiTFSI with the polymer matrix, however, differ substantially depending on the polymer host. In the case of PVDF‐HFP, SN and LiTFSI do not alter its crystallinity, and the system remains a physical mixture of two distinct phases. As a result, the ionic conductivity depends largely on the composition of SN and LiTFSI [12]. In contrast, SN forms a eutectic mixture with PEO and LiTFSI, which inhibits PEO crystallization and lowers its melting point [20]. Upon addition of LiTFSI, the salt is partially dissolved in SN and partially complexed with PEO, with PEO and SN exhibiting weak interactions [12]. This behavior resembles that of gel electrolyte systems and offers a new pathway for developing non‐flammable solid‐polymer electrolytes. The PEO‒SN‒LiTFSI ternary system exhibits a range of coexisting crystalline, isotropic noncrystalline, and liquid regions depending on the composition and temperature [18, 20]. Crystalline domains contribute to mechanical integrity, while isotropic noncrystalline and liquid regions promote high ionic conductivity; therefore, achieving an optimal balance among these phases is crucial. Additionally, the homogeneous formation of ion‐conducting channels throughout the PEO matrix has been reported to be critical for fast lithium‐ion transport [21]. Owing to the close size match between SN molecules and the ─O─CH_2_─CH_2_─O─segments in PEO, SN forms continuous Li^+^ transport channels, effectively decoupling ion conduction from the conventional complexation/decomplexation mechanism involving ether oxygen (EO) [22]. Therefore, in addition to addressing the mechanical and chemical instabilities of PEO‒SN‐based solid polymer electrolytes, ensuring homogeneous channel formation should also be considered a key design criterion.

A high SN content (SN:EO = 1:4) combined with a low salt concentration (Li^+^:EO = 1:32) strikes an optimal balance, enabling the formation of homogeneous conducting channels and a free‐standing membrane with reasonable ionic conductivity [21]. When paired with lithium iron phosphate (LFP) and lithium electrodes, this solid‐state membrane delivers high capacity and stable cycling performance at both low and room temperatures. However, its performance deteriorates rapidly at 50°C. This degradation may be associated with the expansion of the isotropic liquid region [21], which can promote electrolyte leakage and intensified parasitic side reactions between SN and lithium at elevated temperatures [15]. Approaches such as confining free SN within polymer fiber networks using additional cross‐linked polymer matrices have shown promise in enhancing mechanical strength and enabling stable battery performance [13, 14, 16]. However, these methods have proven effective only at low and ambient temperatures. Considering the composition‐dependent shift in melting point and the associated temperature‐dependent changes in the isotropic liquid phases‒along with the fact that batteries operate over a wide temperature range [23, 24] and are subject to the formation of localized thermal hotspots [25]‒it is essential to investigate the temperature‐dependent behavior of SN‐related reactions and to develop comprehensive solutions that address challenges associated with SN‐based electrolytes across a broad temperature window.

Here, our study reveals that the interfacial reactions of SN accelerate with increasing temperature. Contrary to the general view, we identify temperature‐dependent reactions of SN at the cathode interface, which contribute substantially to interfacial degradation and capacity fading, underscoring the need to protect both electrodes. A moderate salt concentration (Li^+^:EO = 1:18) and high SN content (SN:EO = 1:4) resulted in a predominantly amorphous electrolyte with trace residual crystallinity and sufficient ionic conductivity. The associated loss in mechanical strength was mitigated by reinforcing the membrane with a PVDF‐HFP nanofibrous layer. On one side of the electrolyte membrane, a PEDOT‐based nanofibrous mat (P‐NFM) coated with a thin fluoroethylene carbonate (FEC)‐containing gel layer further enhanced mechanical stability without compromising ionic conductivity, effectively protecting the lithium anode even as 50°C. Meanwhile, LFP cathode particles were protected by a sulfonate‐functionalized PEDOT (PEDOT‐S) coating. The resulting free‐standing, multilayer electrolyte membrane, paired with the PEDOT‐S‐coated LFP cathode, delivered stable electrochemical performance over a temperature range from 10°C to 50°C.

## 2 Results and Discussion

### 2.1 Inter‐Relations Between Composition, Temperature, and Chemical Reactivity

The PEO‒SN‒LiTFSI solid polymer electrolyte must satisfy four critical requirements: mechanical integrity, chemical stability, homogeneous ion‐conducting channel formation, and sufficient ionic conductivity. This ternary system comprises coexisting crystalline, isotropic noncrystalline, and liquid phases whose relative fractions depend on composition and temperature. Therefore, precise compositional control is essential for tuning these properties and understanding the associated phase behavior and chemical reactivity. A fundamental trade‐off exists: mechanical strength arises from crystalline domains, whereas high ionic conductivity requires amorphous or isotropic liquid regions. The formation of homogeneous conducting channels is further governed by the SN:EO ratio [21]. Therefore, we fixed the SN:EO molar ratio at 1:4 and varied the Li^+^:EO molar ratio (1:32, 1:18, and 1:12) to evaluate phase evolution and identify the optimal composition.

Thermogravimetric analysis (TGA) of the prepared membranes confirmed the intended composition, showing an SN mass loss of 23%–25% across all samples (Figure S1). X‐ray diffraction (XRD) patterns of PEO‒SN and PEO‒LiTFSI binary mixtures exhibited strong PEO crystalline peaks that progressively diminished with increasing salt content, indicating an increase in isotropic noncrystalline regions (Figure 1A). In the ternary membranes, PEO crystallinity decreased further, with the characteristic crystalline peaks disappearing at Li^+^:EO = 1:18, suggesting near‐complete amorphization. Differential scanning calorimetry (DSC) thermograms further supported this crystalline‐to‐amorphous transition, with multiple melting peaks appearing at temperatures lower than those of pure PEO (64°C) and SN (58°C) in the binary mixtures (Figure 1B) [20]. In the ternary mixtures, these melting features shifted to even lower temperatures and disappeared entirely at Li^+^:EO = 1:12, indicating the formation of a fully isotropic amorphous structure.

**FIGURE 1:**
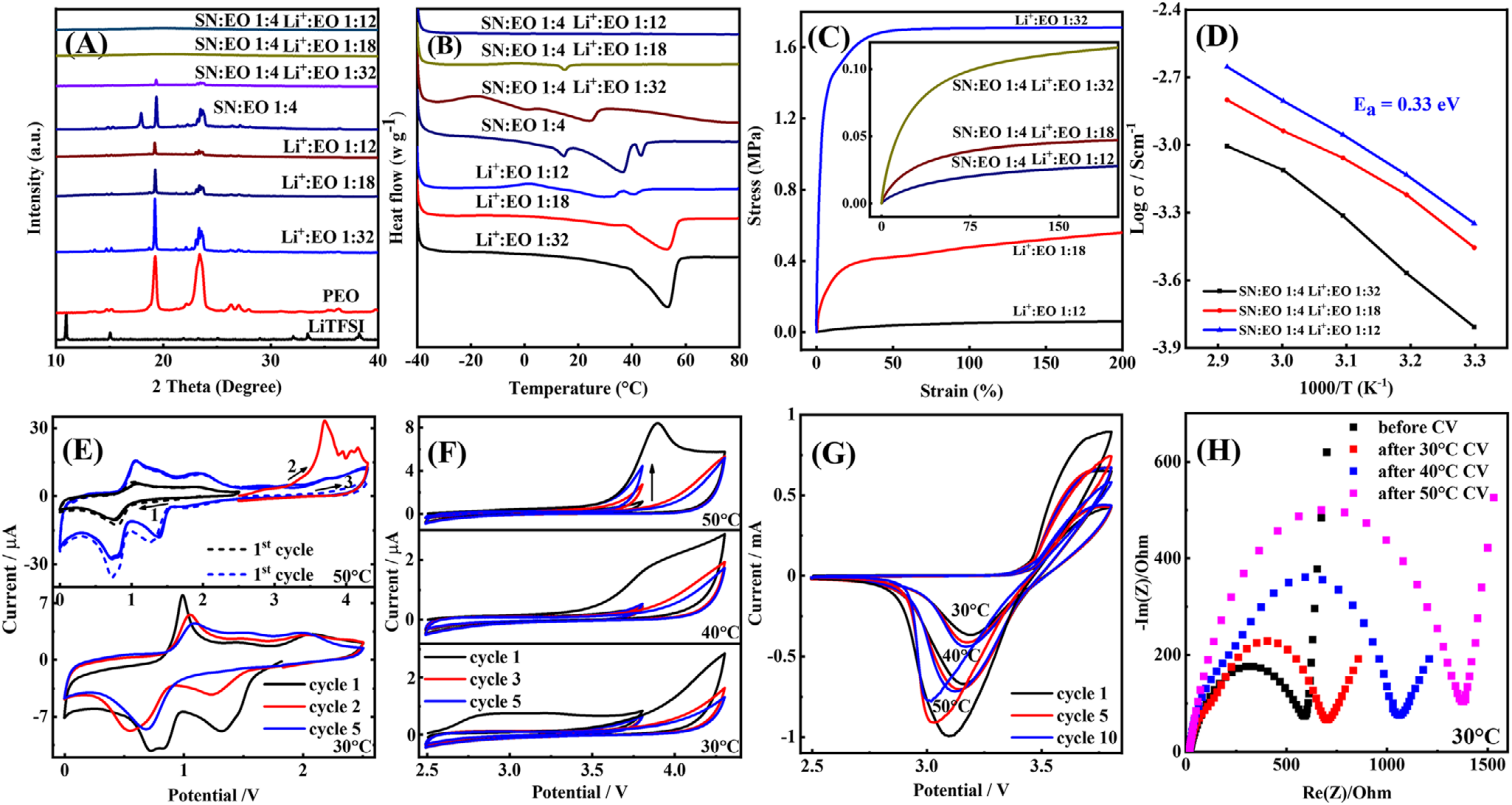
Composition‐ and temperature‐dependent properties and interfacial reactions of PEO‒SN‒LiTFSI electrolytes. (A) XRD patterns and (B) DSC thermograms showing composition‐dependent structural and thermal changes in polymer membranes. (C) Stress–strain curves demonstrating the corresponding changes in mechanical strength. (D) Arrhenius plots showing the increase in ionic conductivity with increasing salt concentration. (E, F) CV profiles of Li|TM14118|SS cells revealing temperature‐driven interfacial reactions. (G) CV profiles and (H) EIS spectra of Li|TM14118|LFP cells showing SN‐associated interfacial reactions at the LFP interface and the associated increase in cell resistance.

These structural transitions resulted in distinct morphological and mechanical differences. Low‐salt membranes (Li^+^:EO = 1:32) were opaque, white, and free‐standing, whereas high‐salt membranes (Li^+^:EO = 1:12) became transparent, soft, and sticky, with substantially reduced mechanical integrity (Figure S2). SEM revealed a corresponding transition from rough to uniformly smooth surface morphologies as salt content increased (Figure S3). Accordingly, mechanical strength declined sharply with decreasing crystallinity, from 1.5 to 0.05 MPa in the binary systems. Incorporation of SN further weakened mechanical robustness, rendering the Li^+^:EO = 1:18 membrane marginally processable and the Li^+^:EO = 1:12 membrane too soft to handle (Figure 1C and Figure S2).

In contrast, ionic conductivity increased with salt concentration, rising from 0.16 to 0.45 mS cm^−^^1^ as the Li^+^:EO ratio increased from 1:32 to 1:12 at 30°C, consistent with the reported values (Figure 1D and Figure S4) [12, 21]. However, the improvement between Li^+^:EO = 1:18 (0.35 mS cm^−^^1^) and Li^+^:EO = 1:12 was modest, despite the pronounced loss of structural integrity. These results show that salt concentration governs the melting behavior and phase transitions of PEO‒SN‒LiTFSI and thereby influences its mechanical and ion‐conducting properties. Given the limited conductivity gains and poor processability at high salt content, Li^+^:EO = 1:18‒with trace residual crystallinity‒was selected for further investigation.

The chemical stability of the selected membrane (TM14118, Li^+^:EO = 1:18) was investigated by cyclic voltammetry using a stainless‐steel working electrode and lithium counter/reference electrode (SS|TM14118|Li). During the initial cathodic sweep from open‐circuit voltage to 0 V at 30°C, two broad reduction features consisting of adjacent peaks at 1.47/1.31 V and 0.83/0.73 V were observed, followed by oxidation peaks at 0.99, 1.48, and 2.05 V during the anodic sweep (Figure 1E). The first reduction pair gradually disappeared, while the second pair initially shifted toward more negative and subsequently toward more positive potentials during the following cycles. Component‐isolation analysis using DEC‒LiTFSI, DEC‒SN‒LiTFSI, and PEO‒LiTFSI electrolytes was performed to resolve the contribution of the individual components to this complex peak behavior (Figure S5). The presence of reduction peaks within similar potential regions in both SN‐free and PEO‐free electrolytes shows that LiTFSI undergoes reduction and contributes to interphase formation. However, upon addition of SN, the broad multiple reduction features at ∼1.3‒1.5 V in DEC‒LiTFSI evolved into a distinct peak at ∼1.6 V, while the distinct ∼0.8 V peak broadened and subsequently shifted toward negative potentials upon cycling, resembling the evolution observed for TM14118. These changes support the participation of SN in the reduction process, indicating that the complex cathodic response of TM14118 arises from overlapping reduction processes involving LiTFSI and SN. The evolution of both reduction regions upon SN addition further suggests SN reduction through radical‐anion formation followed by subsequent polymerization reactions.

Increasing the operating temperature of the SS|TM14118|Li cell to 50°C did not alter the overall peak pattern but stabilized the redox features (Figure 1E), suggesting the formation of a redox‐active interphase through overlapping SN and LiTFSI reduction processes within this potential range. When the upper potential limit of the cell was extended to 4.3 V, additional oxidation peaks emerged, including a dominant peak at ∼3.7 V. These peaks largely disappeared when the anodic potential window was subsequently restricted to 2.5 V, indicating irreversible changes in the interface formed at higher potentials. Repeated cycling of the same cell between 0‒4.3 V restored the oxidation response, while the redox profile reverted to that initially observed at 30°C but with substantially higher current intensity, consistent with renewed electrolyte reactions following modification of the previously formed interphase. The enhanced reactivity upon increasing temperature, together with the potential‐dependent evolution of the interphase, shows that these interfacial reactions in TM14118 are strongly governed by both temperature and operating potential.

Evidence for concurrent reactions at the lithium electrode was provided by membrane discoloration after cycling, with color changes observed on both electrode sides (Figure S6), and three‐electrode measurements (Figure S7), which reproduced the same redox peaks with a potential shift (∼0.1 V) consistent with polarization associated with lithium interface formation. Direct thermal contact between lithium metal and the membrane at 60°C produced no significant color change, supporting that the observed discoloration results from concurrent electrochemical reactions (Figure S8).

To identify the origin of the high‐potential oxidation peak observed in TM14118 upon extension of the potential window to 4.3 V, SS|electrolyte|Li cells with TM14118, SN‐free PEO‒LiTFSI, PEO‐free DEC‒SN‒LiTFSI, and PEO/SN‐free DEC‒LiTFSI electrolytes were cycled between 2.5‒3.8 V and 2.5‒4.3 V at 30‒50°C (Figure 1F and Figure S9). The appearance of an oxidation peak above 3.8 V in PEO‒LiTFSI at 50°C indicates that the SN‐free PEO‒LiTFSI electrolyte also undergoes oxidation at higher potential and temperature. The onset of oxidation at ∼3.5 V in DEC‒LiTFSI further demonstrates the contribution of LiTFSI, in addition to PEO, to the oxidation process. Importantly, the addition of SN to DEC‒LiTFSI produced a distinct oxidation peak at ∼4.0 V, confirming the participation of SN in the high‐potential oxidation process. In TM14118, the oxidation current progressively increased with temperature, while extension of the potential window to 4.3 V resulted in the emergence of a pronounced peak above 3.6 V, showing that this oxidation process is governed by both temperature and operating potential (Figure 1F). The persistence of this characteristic peak at lower salt concentration with increasing temperature, together with its substantial attenuation upon decreasing the SN concentration at 50°C, further identifies SN as the major contributor to the distinct high‐potential oxidation peak (Figure S10). The temperature‐dependent increase in oxidation peak intensity reflects accelerated interfacial reaction kinetics at elevated temperature and is also consistent with the reported expansion of the isotropic liquid region with increasing temperature [21], which increases SN mobility and facilitates its transport to the electrode interface during the equilibration period. Increasing the upper potential limit further promotes this oxidation reaction. The rapid attenuation of the oxidation peak in subsequent cycles indicates rapid consumption of reactive species at the interface. Their subsequent replenishment from the bulk electrolyte depends on concentration‐gradient‐driven transport; consequently, the relatively modest oxidation current in subsequent cycles may reflect both depletion of readily reactive interfacial species and their limited replenishment from the bulk electrolyte.

The implications of these reactions were further evaluated using practical electrode materials. Replacing stainless steel with LFP, LTO, and LCO resulted in pronounced temperature‐dependent impedance growth (Figure 1G, H and Figure S11). Among them, LFP exhibited a progressive negative shift in reduction potential together with a substantial increase in cell resistance. In contrast, the SN‐free LFP|PEO–LiTFSI|Li cell exhibited a modest positive shift in reduction potential together with decreasing impedance as the temperature increased (Figure S12). The SN‐free cell initially exhibited relatively high overall resistance with two semicircular responses in the high‐ and medium‐frequency regions, which can be generally associated with bulk electrolyte/contact resistance and interfacial/charge‐transfer processes, respectively [26, 27, 28]. As the operating temperature increased above the melting region of the electrolyte, its ionic conductivity increased by approximately one order of magnitude, from 0.01 mS cm^−1^ at 30°C to 0.1 mS cm^−1^ at 50°C. The resulting improvement in ion transport, electrode‐electrolyte contact, and interfacial kinetics likely contributes to the positive shift in reduction potential and decrease in impedance. In contrast, TM14118 exhibited a negative shift in reduction potential accompanied by substantial impedance growth with increasing temperature. This contrasting behavior between the SN‐containing and SN‐free electrolytes indicates that the additional temperature‐induced impedance growth observed with TM14118 is not an intrinsic response of the LFP|PEO‒LiTFSI interface and supports SN as a major contributor to the additional interfacial resistance developed at the cathode. Similar temperature‐dependent impedance growth was observed for LTO and LCO electrodes, indicating that such electrolyte‐related interfacial instability is not unique to LFP and extends to other practical electrode materials (Figure S11). The substantially greater impedance growth observed with these porous particulate electrodes than with planar stainless steel may also be associated with their larger effective electrolyte‐electrode interfacial contact area and greater number of accessible reactive sites (Figure S13). For example, secondary LFP particles, with a specific surface area of ∼14 m^2^ g^−1^, provide a potentially much larger accessible interface for electrolyte contact than the planar SS electrode, thereby promoting electrolyte decomposition and interphase accumulation that contribute to the observed large impedance growth.

Collectively, these results reveal that interfacial reactions of PEO‒SN‒LiTFSI electrolytes involve overlapping reactions of their components. Both LiTFSI and SN undergo reduction reactions, while SN substantially alters the reduction response and possibly undergoes polymerization. At higher potentials, all three electrolyte components undergo oxidation, with SN being the major contributor to the characteristic ∼3.7 V oxidation feature in TM14118. These interfacial reactions are strongly accelerated by temperature and further influenced by operating potential, electrolyte composition, and effective electrolyte‐electrode interfacial contact area. Concurrent reactions at lithium contribute to interfacial polarization and necessitate anode protection; however, the substantially greater impedance growth observed with practical cathodes identifies cathode‐side reactions as a major contributor to cell degradation. These findings establish temperature‐driven interfacial reactivity, with a significant contribution from SN, as a fundamental limitation of PEO‐based electrolytes with high SN content and demonstrate the need to protect both electrode interfaces.

### 2.2 Multilayer Electrolyte Membrane to Suppress Temperature‐Driven SN‐Lithium Interfacial Reactions

Building on the preceding results which show that the optimized TM14118 electrolyte (Li^+^:EO = 1:18) provides homogeneous ion‐conducting pathways and adequate ionic conductivity but suffers from insufficient mechanical integrity and pronounced temperature‐driven interfacial reactivity with lithium‒a multilayer electrolyte membrane (MLEM14118) was rationally designed to address both limitations (Figure 2A). The multilayer architecture is intended to simultaneously reinforce mechanical robustness and suppress the transport of reactive molecules toward the lithium interface. PVDF‐HFP fibers were employed as the mechanical backbone owing to their high crystallinity, thermal stability, and chemical inertness toward SN. To mitigate temperature‐driven electrolyte transport to the electrode and stabilize the lithium interface, a nanofibrous mat composed of PEO, PEDOT:PSS, and carbon nanotubes (P‐NFM), subsequently coated with a PEO‒LiTFSI‒FEC gel layer, was integrated on the opposite side. In this configuration, the fibrous P‐NFM serves as a physical barrier and adsorption scaffold to confine the electrolyte, while the FEC‐containing gel promotes the formation of a chemically stable and lithium‐compatible interfacial layer. The final free‐standing MLEM14118 membrane was fabricated by sandwiching the TM14118 electrolyte between these two functional polymer networks, followed by solvent removal, as illustrated in Figure 2A.

**FIGURE 2:**
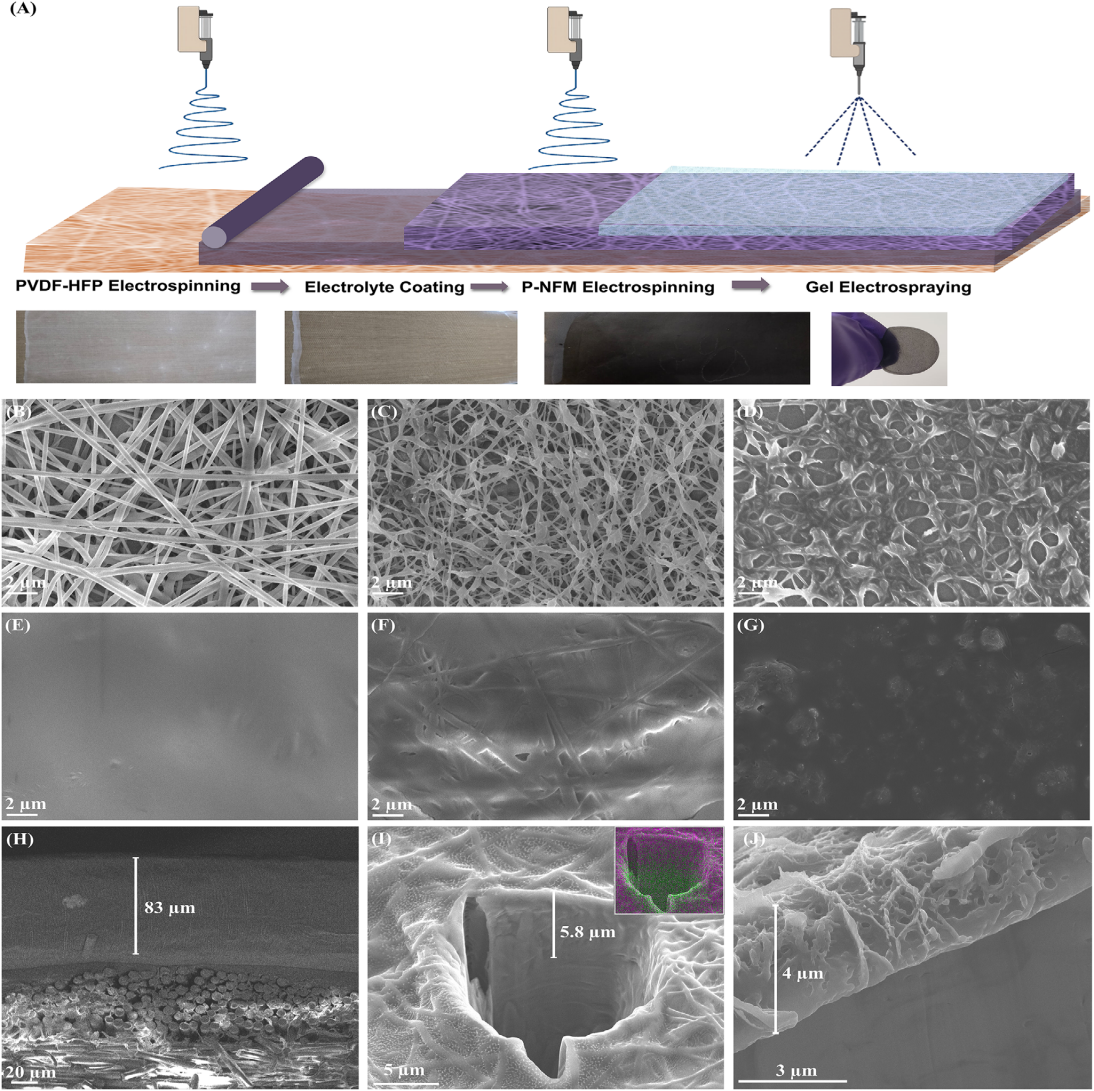
Fabrication and structural characterization of the multilayer MLEM14118 membrane. (A) Schematic and photographs illustrating the stepwise fabrication of MLEM14118. (B, C) SEM images of electrospun PVDF‐HFP and P‐NFM fibrous networks, respectively. (D) SEM image showing conformal gel coverage of the P‐NFM network after electrospraying. (E, F) Top and bottom SEM images of the TM14118‐infused PVDF‐HFP layer, showing electrolyte infiltration into the fibrous scaffold. (G) SEM image of the P‐NFM layer electrospun onto the infused electrolyte surface. (H–J) Cross‐sectional SEM and FIB depth profiling of MLEM14118, confirming the multilayer architecture and electrolyte infiltration.

PVDF‐HFP was first electrospun onto a Teflon substrate, forming a porous fibrous network with an average fiber diameter of ∼300 nm (Figure 2B). Casting the electrolyte onto this network produced a smooth top surface (Figure 2E), whereas the bottom surface exhibited distinct fiber imprints (Figure 2F), indicating effective electrolyte infiltration into the fibrous scaffold. Focused ion beam (FIB) cross‐sectioning revealed that the electrolyte‐infused PVDF‐HFP layer extended to a depth of approximately 5.8 µm above the smooth bulk electrolyte layer. This penetration depth was further corroborated by energy‐dispersive x‐ray (EDX) mapping, where variations in fluorine intensity between the slightly irregular electrolyte‐infused fibrous region and the smooth continuous bulk electrolyte confirmed successful fiber infiltration and the formation of a mechanically supporting layer (Figure 2I).

On the opposite side of the electrolyte, electrospinning of the P‐NFM resulted in micron‐scale agglomerates partially submerged within the electrolyte matrix, distinctly different from the fiber imprint morphology observed for PVDF‐HFP (Figure S14). Subsequent electrospraying of the gel layer yielded the complete MLEM14118 membrane with an overall thickness of ∼83 µm (Figure 2H). The gel coating uniformly covered the agglomerated surface features, forming a smooth and continuous thin polymer layer (Figure 2G).

To elucidate the origin of the agglomerated morphology, P‐NFM was electrospun independently onto a Teflon substrate, producing a nonuniform fibrous network with an average fiber diameter of ∼240 nm (Figure 2C). Electrospraying the gel onto this network resulted in pore filling and conformal coverage of the fibers (Figure 2D). Correlating these observations with the submerged agglomerates in the full membrane suggests that the P‐NFM partially infiltrated the electrolyte during fabrication, while the gel layer remained predominantly on top, forming a distinct interfacial layer. Cross‐sectional analysis confirmed this layered structure, revealing a ∼4 µm thick P‐NFM/gel layer clearly distinguishable from the underlying bulk electrolyte (Figure 2J).

Electrolyte infusion into the PVDF‐HFP fibrous scaffold significantly enhanced the mechanical robustness of the membrane, increasing the tensile strength from 0.05 MPa (pristine TM14118) to 0.24 MPa. Incorporation of the gel‐infused P‐NFM layer further increased it to 0.30 MPa, showing that the dual polymer networks effectively reinforce the membrane (Figure 3A). Figure 3B shows the temperature‐dependent ionic conductivity of the membranes. Notably, electrolyte infusion into the PVDF‐HFP scaffold did not alter the room‐temperature ionic conductivity, which remained at 0.34 mS cm^−^^1^, consistent with the non‐interactive nature of PVDF‐HFP toward the SN‐containing electrolyte. Although the introduction of the relatively dense P‐NFM interlayer slightly reduced the conductivity to 0.29 mS cm^−^^1^, subsequent gel infusion restored it to its original value (Figure S15). Thus, MLEM14118 maintains homogeneous ion‐conducting channels and good ionic conductivity together with improved mechanical stability.

**FIGURE 3:**
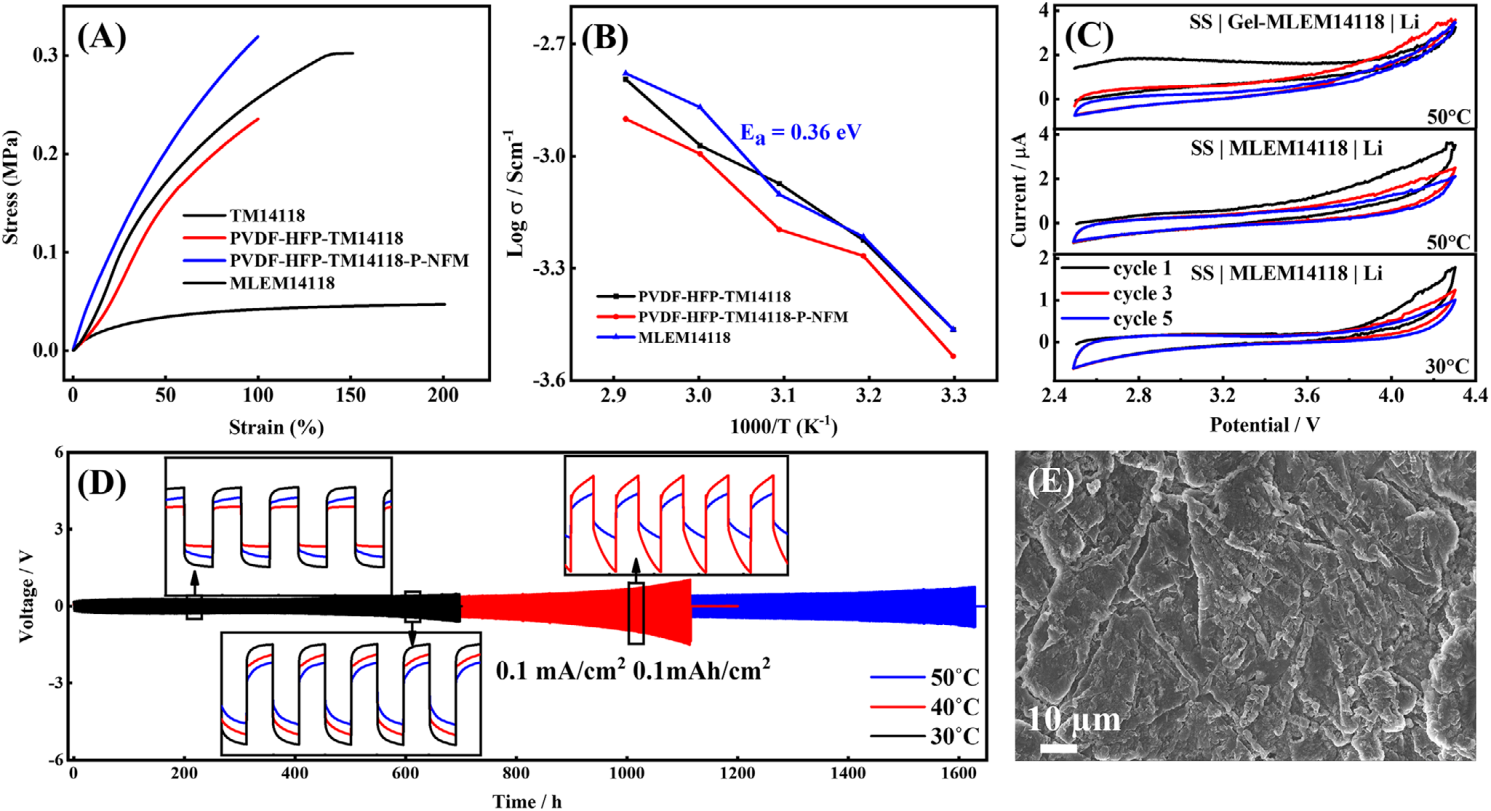
Mechanical, ion‐transport, and lithium‐interface properties of the MLEM14118 membrane. (A) Stress‐strain curves showing progressive enhancement in tensile strength with sequential layer integration. (B) Arrhenius plots showing changes in ionic conductivity upon addition of each layer. (C) CV profiles of lithium|electrolyte|stainless steel cells, demonstrating suppression of temperature‐driven interfacial reactions by MLEM14118. (D) Li|Li symmetric cell cycling, demonstrating stable lithium plating/stripping across different temperatures. (E) SEM image of the lithium surface after 100 cycles at 50°C, showing flake‐like and granular deposits without needle‐like dendrites.

The ability of MLEM14118 to suppress temperature‐driven interfacial reactions at the lithium interface was evaluated using stainless steel|MLEM14118|lithium cells, with the P‐NFM/gel layer facing the lithium electrode (Figure 3C). In contrast to TM14118, which exhibited a pronounced oxidation peak centered at ∼3.7‒3.9 V at elevated temperature, MLEM14118 displayed only a broad oxidation peak above 3.8 V resembling that of the SN‐free PEO‒LiTFSI electrolyte. Although the oxidation current increased slightly with temperature, the characteristic high‐temperature SN‐associated oxidation peak observed with TM14118 was absent. The disappearance of the 3.7‒3.9 V oxidation peak with the introduction of P‐NFM/gel at the lithium interface and the PVDF‐HFP scaffold at the stainless‐steel interface provides additional insight into the reaction mechanism. Combined with the earlier observation of concurrent reactions at lithium, the absence of this peak suggests that both restricted transport of reactive molecules toward the electrode interfaces and suppression of lithium‐side reactions and associated crosstalk contribute to the observed oxidation behavior [16].

Further suppression of the oxidation response was observed when the gel layer was introduced onto the PVDF‐HFP scaffold facing the stainless‐steel side (gel‐MLEM), indicating that the gel layer acts as a transport‐regulating barrier that limits the transport of reactive molecules toward the electrode interfaces and reduces the local accumulation of reactive species. Nevertheless, the characteristic reduction and oxidation features reappeared when the potential window was extended to 0‒4.3 V, even when both electrodes were protected by the gel‐coated PVDF‐HFP scaffold and P‐NFM layer (Figure S16). This result indicates that the membrane architecture cannot completely eliminate potential‐ and temperature‐assisted transport of reactive species driven by concentration gradients within the electrolyte. These findings indicate that the gel‐coated P‐NFM interlayer effectively suppresses electrolyte reactions at the lithium interface and the resulting electrode crosstalk, but that a simple gel‐layer modification remains insufficient to fully prevent interfacial reactions at porous cathodes with extended electrolyte‐electrode contact areas. This limitation highlights the need for an additional cathode‐protection strategy to suppress electrolyte‐induced interfacial degradation.

The dendrite‐suppressing capability of MLEM14118 was further evaluated in Li|Li symmetric cells, with both sides of the membrane coated with the P‐NFM/gel layer. The cells exhibited stable lithium plating/stripping for over 600 h at all tested temperatures and sustained cycling exceeding 1500 h at 50°C (Figure 3D), indicating uniform lithium deposition and stripping behavior. SEM analysis of the lithium surface after 100 cycles at 50°C verified the absence of needle‐like dendrites and revealed flake‐like and granular lithium deposits under the MLEM14118 membrane (Figure 3E), supporting the suppression of dendritic growth. EDX mapping of the deposited lithium surface showed fluorine signals in addition to carbon and oxygen (Figure S17), suggesting incorporation of interfacial‐layer components into the SEI and their contribution to uniform lithium deposition.

### 2.3 Sulfonated PEDOT‐S Coating to Protect the Cathode Interface

As the previous section demonstrated that MLEM14118 alone cannot fully suppress concentration gradient‐assisted electrolyte reactions at the cathode interface, a particle‐level dual‐conducting interfacial layer was developed to protect the porous LFP cathode with its extended electrolyte–electrode interfacial contact area. The design simultaneously enables Li^+^ transport across the electrode‐electrolyte interface and electronic conduction throughout the cathode network. PEDOT was selected because of its high electronic conductivity and proven interfacial stability at elevated temperatures, while sulfonate functionalization was introduced to provide ionic conductivity and improve aqueous processability [29, 30, 31, 32, 33, 34, 35, 36, 37, 38]. A schematic illustration of the synthesized PEDOT‐S and its conformal coating on LFP particles is shown in Figure 4A.

**FIGURE 4:**
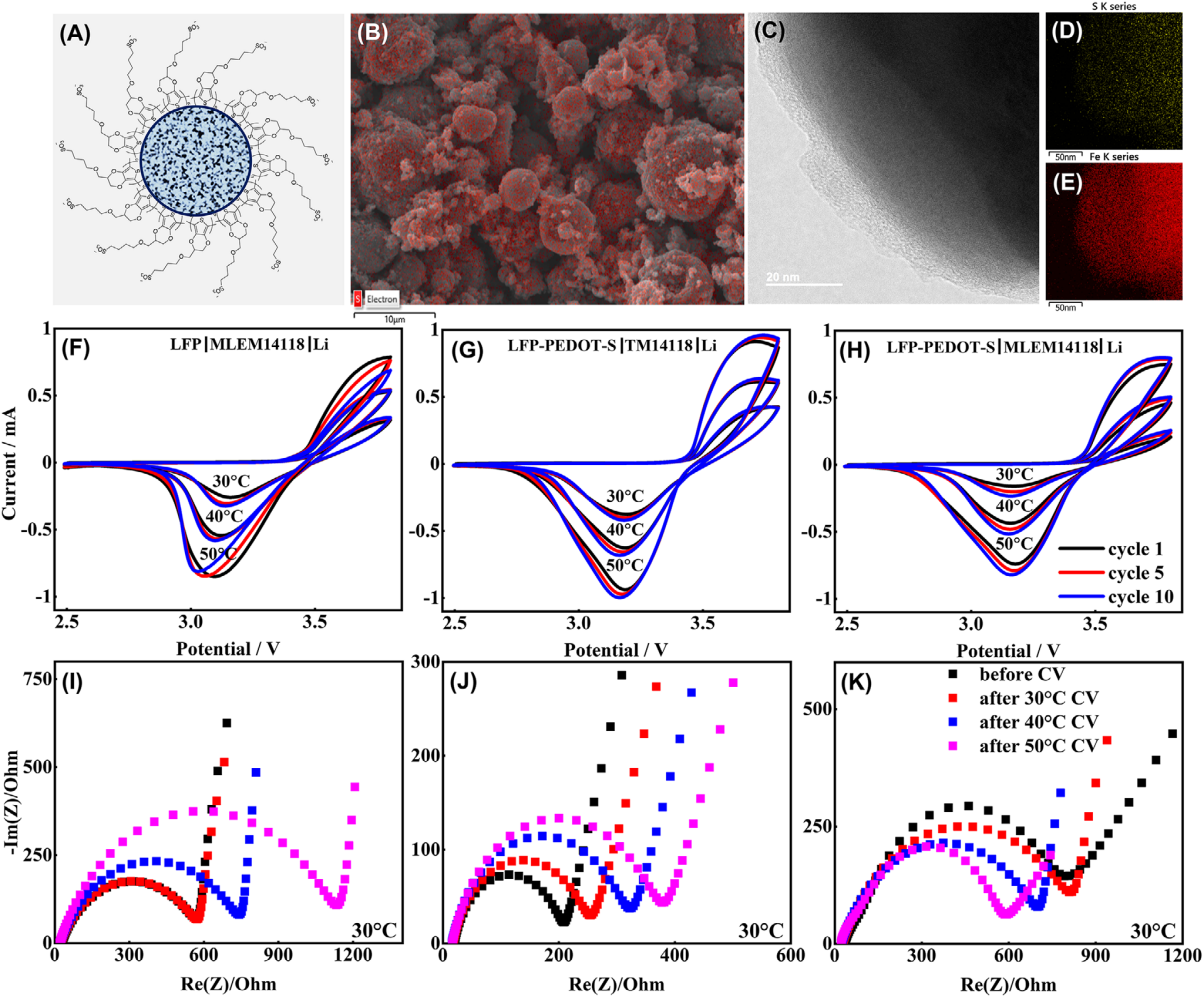
PEDOT‐S coating and stabilization of the LFP‐electrolyte interface. (A) Schematic illustration of sulfonate‐functionalized PEDOT coating on LFP particles. (B) SEM image with EDX sulfur mapping, showing uniform polymer coverage. (C–E) TEM images and elemental mapping revealing conformal PEDOT‐S coating. (F–H) CV profiles and (I‐K) corresponding EIS spectra of cells with pristine LFP and PEDOT‐S‐coated LFP paired with TM14118 and MLEM14118, demonstrating suppression of temperature‐driven cathode interfacial reactions and associated resistance evolution.

Successful PEDOT‐S coating was confirmed by FTIR through the characteristic sulphonate stretching vibration at 1200 cm^−1^ (Figure S18). SEM imaging and sulfur elemental mapping indicated uniform coating over secondary LFP particles (Figure 4B). TEM imaging of the coated particles revealed a conformal amorphous shell with an average thickness of 5‒7 nm (Figure 4C), which likely arises from a combination of the native carbon coating on LFP and the PEDOT‐S layer. The sulfur distribution around the iron core in elemental mapping supports homogeneous particle‐level polymer coverage and the incorporation of PEDOT‐S within the observed amorphous shell (Figure 4D, E).

To evaluate the protective capability of the PEDOT‐S coating and to decouple the role of individual electrode‐electrolyte interfacial reactions, cells were assembled using pristine LFP with MLEM14118, and PEDOT‐S‐coated LFP with both TM14118 and MLEM14118 membranes. The CV response of pristine LFP paired with MLEM14118 resembled the behavior observed with TM14118, showing a progressive negative shift in the reduction peak with increasing temperature (Figure 4F), accompanied by a significant increase in cell resistance (Figure 4I), consistent with continued SN‐associated interfacial reactions at the unprotected cathode. In contrast, PEDOT‐S‐coated LFP reversed this trend, even when paired with TM14118, exhibiting a stable and well‐defined reduction peak with a slight positive shift and minimal temperature dependence (Figure 4G). However, the overall cell resistance still increased, consistent with continued interfacial reactions at the lithium anode (Figure 4J).

When both interfaces were protected using the MLEM14118 membrane with PEDOT‐S coating on LFP, the total cell resistance decreased with increasing temperature (Figure 4K), while the reduction peak position remained stable across all temperatures (Figure 4H). The initially higher resistance, arising from multiple interfacial layers, gradually decreased upon cycling, likely due to improved ionic transport and evolution of the PEDOT‐S‐containing interface. To further understand this resistance evolution, PEDOT‐S‐coated LFP was evaluated with the SN‐free PEO‒LiTFSI electrolyte (Figure S19). Similar to pristine LFP with PEO–LiTFSI, the reduction potential shifted positively with increasing temperature, while the reduction peak remained stable without negative potential drift. The high initial impedance of PEDOT‐S‐LFP|PEO‒LiTFSI|Li decreased after cycling at 30°C, with a more pronounced decrease after cycling at 50°C. The greater decrease compared with pristine LFP|PEO‒LiTFSI|Li indicates that, in addition to temperature‐dependent changes in the PEO‒LiTFSI electrolyte, evolution of the PEDOT‐S‐containing interface likely contributes to the reduction in cell resistance.

The similarity of this impedance evolution to that of PEDOT‐S‐LFP|MLEM14118|Li suggests combined contributions from improved ionic transport through MLEM14118 at elevated temperature and evolution of the PEDOT‐S‐containing interface. These results show that SN‐associated interfacial reactions contribute substantially to the temperature‐dependent cathode impedance growth, and confirm that the conformal PEDOT‐S coating establishes a dual‐conducting interfacial layer that effectively suppresses these reactions, highlighting the necessity of protecting both electrodes for stable operation.

### 2.4 Electrochemical Performance of the Dual‐Side Protected Cells

The shifts in LFP reduction peak potentials and the corresponding impedance evolution observed for protected and unprotected interfaces at elevated temperatures are reflected in the long‐term cycling performance of cells assembled with pristine LFP and PEDOT‐S‐coated LFP, paired with TM14118 and MLEM14118 membranes (Figure 5). Cells assembled with pristine LFP and TM14118 rapidly lost capacity within 50 cycles, consistent with the previous observation (Figure 5A) [20]. Unrestricted interfacial reactions at both electrodes increased cell resistance, leading to rising overpotential during cycling (Figure S20A) and continuous lithium consumption, resulting in reduced coulombic efficiency. Replacing TM14118 with MLEM14118 did not mitigate capacity fading, as the cathode remained unprotected; however, the initial coulombic efficiency improved slightly, reflecting stabilization of the lithium interface. The overpotential continued to increase during cycling (Figure S20B), consistent with persistent SN‐associated interfacial reactions at the cathode. In contrast, protection of the cathode using PEDOT‐S significantly improved capacity retention and reduced overpotential, in agreement with the stabilized interfacial response observed by CV and impedance measurements. However, the cell still lost ∼40% capacity over 250 cycles, accompanied by a gradual decrease in coulombic efficiency and a marked increase in overpotential associated with continued interfacial reactions at the unprotected lithium anode (Figure S20C). Thus, although cathode protection substantially improves cycling stability, degradation at the remaining unprotected lithium interface continues to limit cell performance.

**FIGURE 5:**
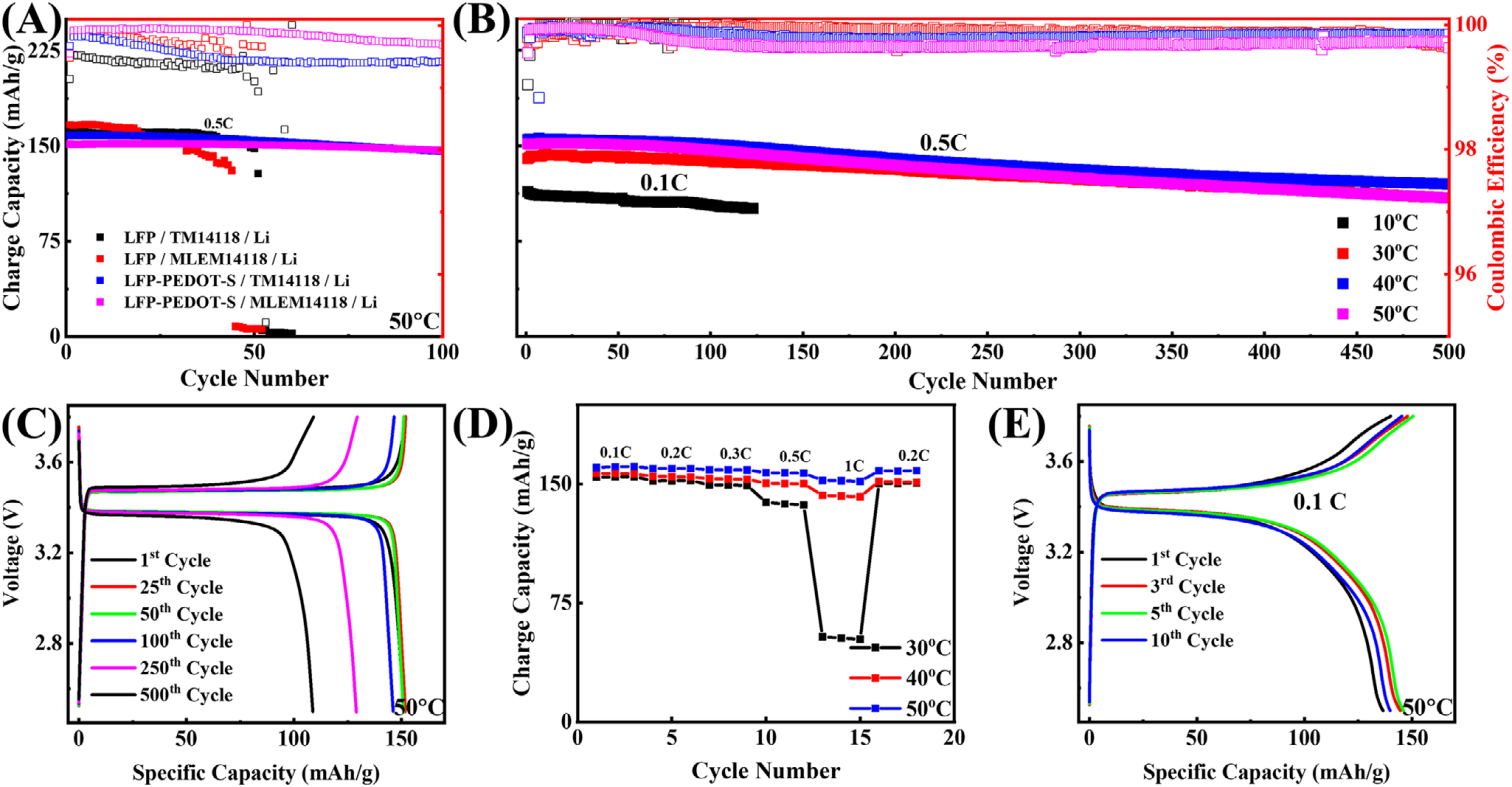
Electrochemical performance of dual‐side protected cells across a wide temperature range. (A) Cycling performance of pristine LFP and PEDOT‐S‐LFP paired with TM14118 and MLEM14118, showing the effects of cathode and lithium‐interface protection on cycling stability. (B) Long‐term cycling of PEDOT‐S‐LFP with MLEM14118 at 10°C–50°C, demonstrating wide‐temperature stability. (C) Charge–discharge profiles at 50°C showing minimal overpotential change over prolonged cycling. (D) Rate performance at different temperatures, indicating effective ion transport through MLEM14118. (E) Electrochemical performance of a PEDOT‐S‐LFP|MLEM14118 pouch cell, demonstrating the feasibility of the integrated cell configuration.

The stabilizing effect of PEDOT‐S on the cathode interface was further examined using the SN‐free PEO–LiTFSI electrolyte. Pristine LFP|PEO‒LiTFSI|Li lost capacity within ∼50 cycles at 50°C (Figure S21). This indicates that the LFP interface remains unstable during prolonged cycling even in the absence of SN, despite the decrease in impedance observed after high‐temperature CV, consistent with the contribution of PEO and LiTFSI to the oxidation process identified by component‐isolation analysis. In contrast, the PEDOT‐S‐LFP|PEO‒LiTFSI|Li cell gradually reached stable cycling after several cycles, accompanied by temperature‐dependent improvements in ion transport, electrode‐electrolyte contact, and interfacial kinetics in PEO‒LiTFSI, together with the impedance evolution associated with the PEDOT‐S‐containing interface. Stable performance of this cell in the absence of SN further indicates that PEDOT‐S also protects the LFP interface against reactions involving the PEO‒LiTFSI electrolyte. However, the prolonged stabilization and lower capacity compared with the PEDOT‐S‐LFP|TM14118|Li cell at 50°C, together with the severely limited capacity utilization at 30°C even at a low C‐rate, indicate restricted ionic transport arising from the low ionic conductivity of the PEO‒LiTFSI electrolyte. Thus, while incorporation of SN establishes homogeneous ion‐conducting channels and substantially enhances ionic conductivity, it simultaneously introduces additional temperature‐dependent interfacial reactions at both electrode interfaces. Therefore, achieving the transport advantages provided by SN while maintaining stable cycling requires simultaneous protection of both electrode interfaces.

When both electrodes were protected using PEDOT‐S‐coated LFP and the MLEM14118 membrane, the cell retained ∼70% capacity over 500 cycles with negligible change in overpotential, demonstrating effective suppression of temperature‐driven interfacial reactions (Figure 5B, C). Although the initial coulombic efficiency decreased slightly during cycling, it remained above 99.6%. At lower temperatures (40°C and 30°C), capacity fading was further reduced, and coulombic efficiency remained high during stable long‐term cycling. Notably, the cell also operated at 10°C for over 100 cycles, indicating robust performance across a wide temperature range despite the presence of multiple interfacial layers. The slight decrease in coulombic efficiency at elevated temperatures suggests minor SN permeation through the protective layers under prolonged operation. The applicability of the dual‐protection strategy at increased material loading was further evaluated using an LFP electrode with a loading of ∼5 mg cm^−2^. After initial stabilization, the cell maintained stable cycling with a specific capacity of ∼152 mAh g^−1^ at 0.3C (Figure S22), showing that the interfacial protection remains effective at increased active‐material loading. The dual‐sided protected cell also exhibited good rate capability, retaining over 90% capacity at 1C at 40°C and 50°C, indicating effective ion transport through the multilayer structure. At 30°C, comparable performance was maintained up to 0.5C, suggesting some transport limitation at lower temperatures. To further evaluate the applicability of the interfacial protection strategy to higher‐voltage cathodes, LCO cells with and without dual protection were operated to an upper cutoff voltage of 4.3 V. The unprotected LCO|TM14118|Li cell lost ∼63% of its capacity within 30 cycles, whereas LCO‐PEDOT‐S|MLEM14118|Li retained 97% of its capacity and continues to cycle stably (Figure S23). The pronounced difference between the protected and unprotected LCO cells demonstrates that the dual interfacial protection strategy effectively protects the higher‐voltage cathode and extends its applicability beyond LFP cathodes. Finally, the MLEM14118 architecture was integrated via stepwise coating onto PEDOT‐S‐coated electrodes to fabricate a pouch cell, which delivered a discharge capacity of ∼1.96 mAh at 0.1C, corresponding to an areal capacity of ∼0.22 mAh cm^−2^. These results collectively demonstrate that dual‐side interfacial protection effectively suppresses temperature‐driven SN interfacial reactions, enabling stable long‐cycle performance across a wide temperature range (10°C‒50°C), and extending the strategy to increased cathode loading, higher‐voltage cathodes, and pouch‐cell configurations. Nevertheless, further optimization of electrode loading, high‐voltage cycling durability, and pouch‐cell engineering will be required to translate this interfacial design toward practical cell operation.

### 2.5 Analysis of Unprotected and Dual‐Side Protected Interfaces

The interfacial chemistry after cycling at 50°C was examined by XPS analysis of lithium‐peeled membranes and aluminum‐peeled cathodes, revealing that the protective layers fundamentally alter interphase formation (Figure 6A). The unprotected TM14118/lithium interface cycled between 0–4.3 V exhibits two N 1s components at 397.6 and 398.3 eV, assigned to Li_3_N and polymerized C═N species, respectively [39, 40, 41]. In contrast, the stainless‐steel interface showed an additional peak at 399.3 eV corresponding to residual nitrile groups. The presence of polymeric nitrogen at the lithium interface indicates that SN polymerization is not confined to the cathode surface, while the absence of residual nitrile species together with the formation of Li_3_N suggests more extensive SN transformation at lithium than at stainless steel due to its higher reactivity. These observations are consistent with the membrane discoloration observed during concurrent reaction analysis and support the lithium‐side contribution to the distinctive high‐temperature oxidation behavior through electrode crosstalk.

**FIGURE 6:**
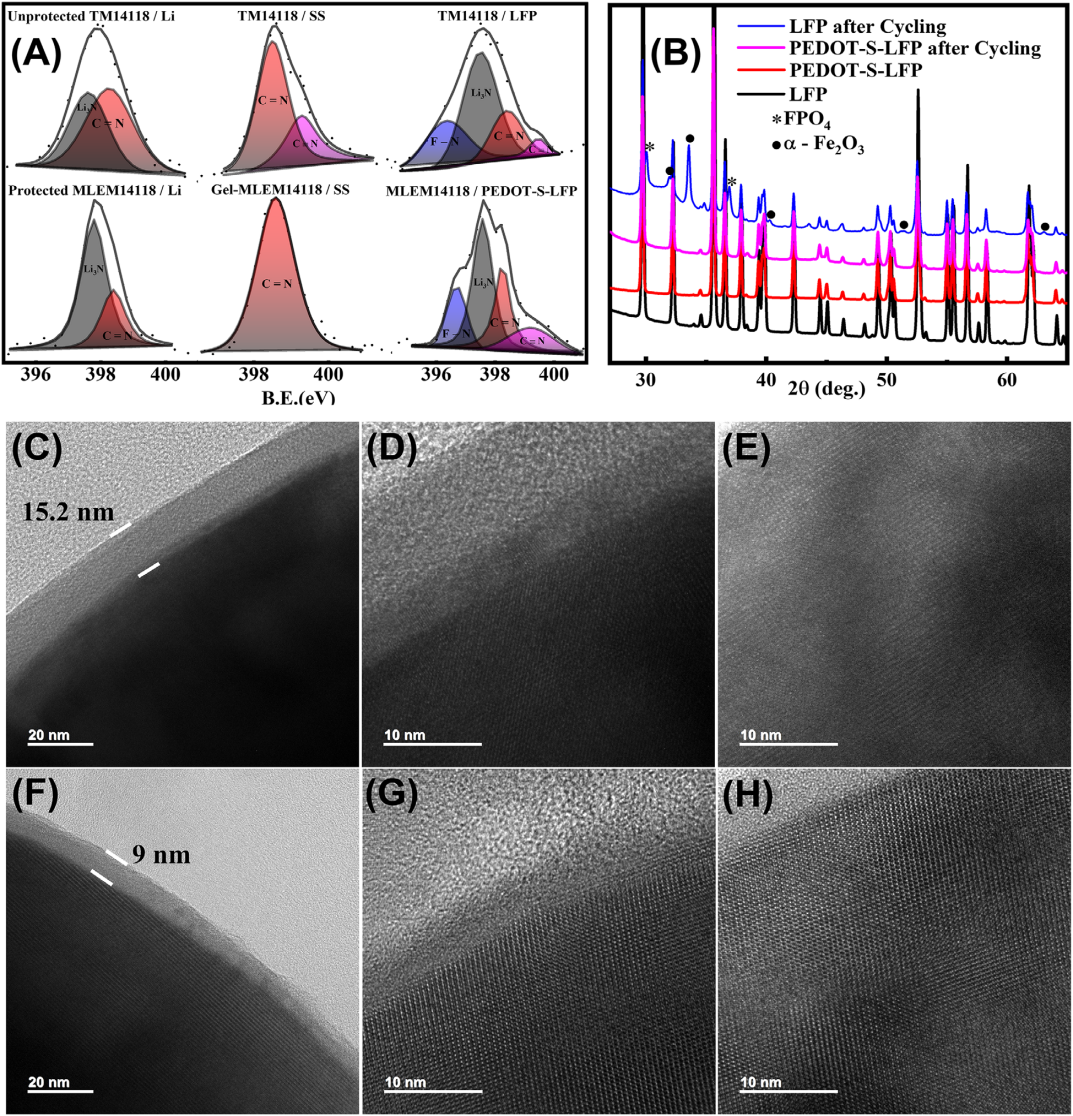
Interfacial chemistry and structural evolution of protected and unprotected electrode interfaces after cycling. (A) XPS spectra of lithium and LFP interfaces after cycling at 50°C, revealing differences in interphase chemistry with and without protection. The N 1s ∼396.4 eV component is tentatively assigned to Fe‒N coordination based on the reported binding energy. (B) Synchrotron XRD patterns showing structural evolution of pristine and PEDOT‐S‐coated LFP before and after cycling. (C–H) TEM images of cycled LFP particles, revealing a thick, heterogeneous interphase and structural degradation in unprotected LFP, in contrast to the thinner, uniform interphase and preserved crystallinity of PEDOT‐S‐coated LFP.

The protected lithium interface exhibited the same N 1s components but with a substantially reduced polymer contribution relative to Li_3_N, indicating that the P‐NFM/gel interface layer effectively suppresses electrolyte transport to the lithium surface and the resulting electrolyte transformation and polymerization reactions. At the PVDF‐HFP‐protected stainless‐steel interface cycled between 2.5–4.3 V, both polymeric and residual nitrile species remained detectable (Figure S24). However, the nitrile signal disappeared upon introduction of the gel layer and expansion of the potential window to 0–4.3 V. These results indicate that the membrane architecture effectively suppresses temperature‐driven electrolyte transport and accumulation but cannot completely prevent concentration‐gradient‐assisted transport of reactive electrolyte species under more aggressive electrochemical conditions.

Replacing stainless steel with a porous LFP cathode having an extended electrolyte–electrode interfacial contact area introduced an additional N 1s component at ∼396.4 eV alongside the Li_3_N, C═N, and nitrile peaks observed at the lithium and stainless‐steel interfaces. Compared with the stainless‐steel interface, the LFP electrode exhibited markedly reduced nitrile intensity together with the emergence of an additional component at ∼396.4 eV, indicating more extensive electrolyte transformation on the high‐surface‐area active cathode material. The additional component is tentatively assigned to Fe─N coordination based on the reported N 1s binding energy at ∼396.4 eV, although confirmation requires complementary Fe spectral analysis [42, 43, 44]. Upon PEDOT‐S coating, the nitrile signal increased significantly while the ∼396.4 eV component decreased, indicating substantial suppression of electrolyte reactions at the LFP surface.

Synchrotron XRD further revealed the structural consequences of these interfacial reactions (Figure 6B). Unprotected LFP underwent severe structural degradation after cycling, evidenced by the emergence of FePO_4_ and α‐Fe_2_O_3_ phases together with a characteristic diffraction peak at 33.55° [45, 46]. In contrast, PEDOT‐S‐protected LFP retained its pristine structure without detectable secondary phases. Correlating these observations with the XPS results suggests that continuous interfacial reactions at the unprotected surface are accompanied by further lithium extraction from LFP, phase transformation and structural instability.

TEM analysis further supported this interpretation. Unprotected LFP develops a thick (∼15.2 nm) heterogeneous amorphous layer (Figure 6C), whereas protected LFP exhibits a thinner (∼8 nm) and more uniform interphase (Figure 6F), closely matching the initial coating thickness. Nitrogen detected across both protected and unprotected particle surfaces supports the involvement of LiTFSI‐ and SN‐derived species in interphase formation (Figure S25). Notably, the thick amorphous layer on unprotected LFP consisted of two distinguishable regions: an outer electrolyte‐derived interphase associated with continuous interfacial reactions and an inner amorphous region arising from structural degradation of LFP (Figure 6D). Consistent with the XRD results, unprotected LFP exhibited discontinuous crystalline domains (Figure 6E), whereas PEDOT‐S‐protected LFP maintained an intact and well‐ordered crystalline framework (Figure 6G, H).

Collectively, these results reveal that continuous interfacial reactions involving the electrolyte components, including SN transformation and polymerization, at unprotected cathode surfaces are accompanied by further lithium extraction from LFP and ultimately by FePO_4_ and Fe_2_O_3_ formation, interphase thickening, and progressive loss of crystallinity. Degradation initiates at the particle surface and propagates into the bulk. In contrast, the PEDOT‐S coating and MLEM14118 membrane act synergistically to suppress reactive electrolyte transport and interfacial reactions, confine interphase growth, and preserve both lithium metal and cathode structural integrity, thereby mitigating the dominant degradation pathway in high‐SN‐content polymer electrolytes.

## 3 Conclusion

This study reveals for the first time that succinonitrile undergoes temperature‐driven interfacial reactions at both electrodes, with cathode‐side reactions constituting the dominant degradation pathway. The composition‐structure‐property interdependence of PEO‒SN‒LiTFSI electrolytes is established, together with the identification of overlapping electrolyte reduction reactions and SN‐associated polymerization at low potentials, oxidative decomposition of multiple electrolyte components with SN as a major contributor at high potentials, and concurrent SN reactions at the lithium interface. A multilayer electrolyte membrane (MLEM14118) suppresses temperature‐driven transport of reactive electrolyte species toward the lithium interface, thereby mitigating lithium‐side reactions and promoting uniform lithium deposition while maintaining homogeneous ion‐conducting pathways and improved mechanical integrity. In parallel, a conformal PEDOT‐S coating on LFP forms a dual‐conducting interfacial layer that protects the cathode against interfacial reactions involving the electrolyte components, including the additional reactions associated with SN. The combined dual‐sided protection stabilizes both electrode interfaces, enabling long‐term cycling (>500 cycles), high coulombic efficiency (>99.6%), and stable operation over a wide temperature range (10°C–50°C). In the absence of protection, continuous electrolyte‐derived interfacial reactions, including SN transformation and polymerization, are accompanied by FePO_4_ and α‐Fe_2_O_3_ formation, interphase thickening, structural degradation, and progressive loss of crystallinity from the particle surface into the bulk. In contrast, the engineered interfacial layers suppress these reactions, confine interphase growth, and preserve electrode integrity. Overall, this work identifies the temperature‐driven interfacial reactivity introduced by SN as a critical trade‐off accompanying its enhancement of ionic conductivity in PEO‐based electrolytes and establishes dual‐sided interfacial engineering as an effective strategy for achieving durable wide‐temperature solid‐state battery operation.

## Author Contributions

**Baskar Selvaraj**: conceptualization, investigation, methodology, validation, formal analysis, data curation, supervision, Writing – original draft. **Hailemichael Ayalew**: methodology, validation. **Yu‐Yao Tsai**: methodology, data curation. **Ly Tho Xuan**: methodology, data curation. **Jia‐Wei She**: software, formal analysis, methodology. **Hao‐Wen Liu**: formal analysis, methodology. **Ming‐Chou Chen**: methodology, supervision. **Yu‐Sheng Hsiao**: conceptualization, methodology, supervision, writing – review and editing. **Nae‐Lih Wu**: methodology, supervision, writing – review and editing. **Hsiao‐hua Yu**: conceptualization, methodology, visualization, project administration, resources, supervision, writing – review and editing.

## Conflicts of Interest

The authors declare no conflicts of interest.

## Supporting information




**Supporting File**: advs78096‐sup‐0001‐SuppMat.docx.

## Data Availability

The data that support the findings of this study are available in the supplementary material of this article.
