# Supplementary material for: Revealing Temperature‐Driven Interfacial Reactions of Succinonitrile in PEO‒LiTFSI Electrolytes and Suppressing Them via Dual PEDOT‐Based Interfacial Layers
Source: Adv Sci (Weinh). 2026 Oct 8:e78096. Online ahead of print. doi: 10.1002/advs.78096 (PMC13650017; doi:10.1002/advs.78096)
Supplement: Supplementary file 1 [file ADVS-9999-e78096-s001.docx]

**Revealing Temperature-Driven Interfacial Reactions of Succinonitrile in PEO‒LiTFSI Electrolytes and Suppressing Them via Dual PEDOT-Based Interfacial Layers**

Baskar Selvaraj^1^, Hailemichael Ayalew^1^, Yu-Yao Tsai^1^, Ly Tho Xuan^2^, Jia-Wei She^1^, Hao-Wen Liu^3^, Ming-Chou Chen^4^, Yu-Sheng Hsiao^2*^, Nae-Lih Wu^3*^, Hsiao-hua Yu^1*^

^1^Smart Organic Materials Laboratory, Institute of Chemistry, Academia Sinica, Taipei 11529, Taiwan

^2^Department of Materials Science and Engineering, National Taiwan University of Science and Technology, Taipei 106335, Taiwan

^3^Department of Chemical Engineering, National Taiwan University, Taipei 10617, Taiwan

^4^Department of Chemistry, National Central University, Taoyuan 32001, Taiwan

^*^Corresponding author at:

**Prof. Yu-Sheng Hsiao:** Department of Materials Science and Engineering, National Taiwan University of Science and Technology, Taipei 106335, Taiwan; E-mail: [yshsiao@mail.ntust.edu.tw](mailto:yshsiao@mail.ntust.edu.tw)

**Prof. Nae-Lih Wu:** Department of Chemical Engineering, National Taiwan University, Taipei 10617, Taiwan; E-mail: [nlw001@ntu.edu.tw](mailto:nlw001@ntu.edu.tw)

**Dr. Hsiao-hua Yu:** Smart Organic Materials Laboratory, Institute of Chemistry, Academia Sinica, Taipei 11529, Taiwan; E-mail: [bruceyu@gate.sinica.edu.tw](mailto:bruceyu@gate.sinica.edu.tw)

**Experimental Section**

**Preparation of PEO‒SN‒LiTFSI Membranes**

Electrolyte membranes were fabricated by solution casting homogeneous mixtures of polyethylene oxide (PEO, Mw = 600 000, Sigma-Aldrich), lithium bis(trifluoromethanesulfonyl)imide (LiTFSI, Sigma-Aldrich), and succinonitrile (SN, Acros Organics) in acetonitrile (ACN). The precursors were dissolved under continuous stirring at 50 ℃ for 12 h and subsequently cast onto Teflon substrates. The resulting films were dried under vacuum at 50 ℃ for 48 h to remove residual solvent. Binary PEO‒LiTFSI membranes were prepared with Li^+^:EO molar ratios of 1:32, 1:18, and 1:12 by mixing 0.408, 0.725, and 1.087 g of LiTFSI with 2 g of PEO, respectively. A binary PEO‒SN membrane was prepared with SN:EO molar ratio of 1:4 by mixing 0.910 g of SN with 2 g of PEO. Ternary PEO‒SN‒LiTFSI membranes were prepared with a fixed SN:EO molar ratio of 1:4 while varying the Li^+^:EO ratios to 1:32, 1:18, and 1:12. The ternary membrane (TM) with SN:EO = 1:4 and Li^+^:EO = 1:18 is denoted as TM14118. In addition, a low-SN ternary membrane was prepared with an SN:EO molar ratio of 1:12 (0.303 g of SN, 0.725 g of LiTFSI, and 2 g of PEO) while maintaining the Li^+^:EO ratio at 1:18. For component-isolation analysis, the same amounts of salt and SN were added to 1g of diethyl carbonate solvent.

**Preparation of Multilayer Electrolyte Membrane (MLEM14118)**

A PVDF-HFP nanofibrous scaffold was first fabricated by electrospinning. PVDF-HFP (Mw = 400 000, Sigma-Aldrich) was dissolved in an acetone/dimethylacetamide mixture (1:2 v/v) to obtain a 14 wt.% solution. Electrospinning was carried out through a 23G needle at a flow rate of 1.0 mL h⁻¹, an applied voltage of 29 kV, and a tip-to-collector distance of 10 cm. A rotating drum collector covered with a Teflon sheet was operated at 50 rpm to obtain a uniform fiber network. The resulting PVDF-HFP mat was dried under vacuum at 120 °C for 2 h. The TM14118 electrolyte solution was then cast onto the PVDF-HFP fiber network and dried at 50 °C for 24 h, allowing complete infiltration of the electrolyte into the porous scaffold.

A PEDOT-based nanofibrous mat (P-NFM) was subsequently electrospun onto the opposite surface using a quaternary blend solution consisting of multi-walled carbon nanotubes (MWCNTs), PEDOT:PSS aqueous dispersion, PEO, and (3-glycidyloxypropyl)trimethoxysilane (GOPS). Electrospinning was performed at a flow rate of 0.3 mL h⁻¹ with a tip-to-collector distance of 12.5 cm. Finally, a gel layer was electrosprayed onto the P-NFM surface from an ACN solution containing PEO, LiTFSI, and fluoroethylene carbonate (FEC) at a molar ratio of 10:1:6, together with 1 vol.% GOPS. The membrane was dried at 50 °C for 24 h. The resulting multilayer electrolyte membrane was denoted MLEM14118 and punched into 19 mm discs for coin-cell testing. An additional gel layer was electrosprayed onto the electrolyte-infiltrated PVDF-HFP side, and the resulting membrane was denoted gel-MLEM14118 for cyclic voltammetry analysis.

**Synthesis of Sulfonated PEDOT (PEDOT-S) and Coating on LFP Particles**

Sulfonated PEDOT (PEDOT-S) was synthesized through a direct C–H arylation polymerization method reported previously by our group. Briefly, a nitrogen-filled Schlenk flask was charged with equimolar amounts of 2,5-diBr-EDOT-S and EDOT-S monomers together with Pd(OAc)₂ (5 mol%) catalyst, P(p-tolyl)₃ (10 mol%) ligand, and Cs₂CO₃ (2 equiv.) base. Anhydrous DMF was added via syringe, and the reaction mixture was stirred at 100 °C for 48 h (Scheme 1). After cooling to room temperature, the reaction mixture was quenched with water. Excess acetone was added to precipitate the product, followed by centrifugation (5000 rpm, 5 min). The precipitate was repeatedly redispersed in water and reprecipitated with acetone until a clear supernatant was obtained. The resulting polymer was further purified by dialysis against deionized water for 2 days using a 1000 g mol⁻¹ cutoff membrane, with water exchanged every 12 h, and subsequently freeze-dried to yield dark blue PEDOT-S powder.

The polymer structure was confirmed by high-temperature ¹H NMR spectroscopy (D₂O, 600 MHz, 90 °C), showing broad peaks at δ 4.47–4.16, 3.63–3.49, 2.55–2.48, and 2.39–2.31 ppm. Molecular weight analysis was performed using a Shimadzu LC-2030C Plus liquid chromatograph equipped with a refractive index detector and a PSS column (PSS MCX 10 µm, 8 × 300 mm, 100 000 Å), calibrated against poly(styrenesulfonic acid sodium salt) standards, yielding M_n_ = 9.8 kg mol⁻¹ and PDI = 1.51.

**Scheme 1.** Synthesis of PEDOT-S through the direct C‒H arylation method.

PEDOT-S coating on LFP or LCO particles was carried out by dissolving PEDOT-S at 2 wt% relative to the mass of cathode material in deionized water and mixing the solution with cathode powder. The suspension was stirred at 50 °C for 1 h in an oil bath. The solvent was then removed under reduced pressure, and the resulting material was dried under vacuum at 120 °C for 2 h to obtain uniformly coated PEDOT-S-LFP or PEDOT-S-LCO particles.

**Electrode Preparation and Electrochemical Characterization:**

Cathodes were prepared by mixing LFP or PEDOT-S-coated LFP with Super P®, PEO binder, and LiTFSI in a weight ratio of 80:10:8:2. The resulting slurry was cast onto aluminum foil and dried at 60 °C for 12 h. The active material loading was controlled within 1.5–2.0 mg cm^-2^. For the increased-loading PEDOT-S-coated LFP cathode (~5 mg cm^-2^) and the LCO cathode, these materials were mixed with Super P®, PVDF binder, and LiTFSI in weight ratios of 90:5:3:2 and 85:10:3:2, respectively. Integrated electrodes were fabricated by sequential deposition of the MLEM14118 layers directly onto the PEDOT-S-coated cathode surface. CR2032 coin cells were assembled in an argon-filled glovebox using lithium metal foil as the counter electrode and the solid polymer electrolyte membrane as the separator. The pouch cell was fabricated using a 3 × 3 cm^2^ LFP cathode containing ~13 mg of active material and a 200 µm thick lithium metal foil as the anode. Galvanostatic charge-discharge measurements were performed using a Neware battery testing system at various temperatures and current densities within voltage windows of 2.5–3.8 V and 3.0–4.3 V. Specific capacities were calculated based on the mass of active material using theoretical capacities of 154 and 156 mAh g^-1^ for LFP and LCO, respectively.

Cyclic voltammetry was conducted using a two-electrode configuration with stainless steel, LFP, LTO, or LCO as the working electrode and lithium metal serving as both counter and reference electrodes. Measurements were performed at different temperatures and potential windows at a scan rate of 0.5 mV s⁻¹. Three-electrode CV measurements were carried out using an EL-CELL configuration with an additional lithium metal reference electrode. Electrochemical impedance spectroscopy (EIS) was performed using a PGSTAT128N Autolab workstation over a frequency range of 7 MHz to 1 Hz with an AC perturbation amplitude of 10 mV. Ionic conductivity was determined using stainless-steel blocking electrodes.

**Materials Characterization**

Changes in crystallinity of binary and ternary membranes were analyzed using powder X-ray diffraction (XRD, Ultima IV, Rigaku) with Cu Kα radiation. Differential scanning calorimetry (DSC) and thermogravimetric analysis (TGA) were performed using a Waters TA instruments Q200 and Perkin Elmer Pyris 1 instruments, respectively. Mechanical properties were evaluated using a Shimadzu AGS-10KNG tensile testing system. Morphological characterization was carried out using field-emission scanning electron microscopy (FE-SEM, ULTRA PLUS, Carl Zeiss) and transmission electron microscopy (TEM, JEOL JEM-1200EX II). Cross-sectional analysis of membranes was performed using a focused ion beam scanning electron microscope (FIB-SEM, FEI Nova600 NanoLab). The presence of sulfonate functional groups in PEDOT-S was confirmed by FTIR spectroscopy. Synchrotron XRD measurements of LFP particles before and after cycling were conducted at the National Synchrotron Radiation Research Center (NSRRC), Taiwan. Interfacial chemical analysis of electrodes after cycling was performed using X-ray photoelectron spectroscopy (XPS, PHI 5000 VersaProbe, ULVAC-PHI, Japan).

**Figure S1.** TGA analysis confirming SN content in ternary PEO–SN–LiTFSI mixtures.

**Figure S2.** Photographs showing composition-dependent morphological transition with varying SN and salt content in binary and ternary membranes.

**Figure S3.** SEM images showing the evolution from crystalline to amorphous morphology in binary and ternary membranes with increasing salt content.

**Figure S4.** EIS spectra of ternary membranes sandwiched between stainless steel electrodes, showing increased ionic conductivity with increasing salt content at 30 °C.

**Figure S5.** Component-isolation CV analysis of SS|Electrolyte|Li cells using SN- and PEO-free DEC‒LiTFSI, PEO-free DEC‒SN‒LiTFSI, SN-free PEO‒LiTFSI and TM14118 electrolytes at 30℃, showing LiTFSI reduction and SN- and PEO-associated electrochemical features.

**Figure S6.** Photographs of the stainless-steel (A) and lithium (B) electrodes after cyclic voltammetry measurements, showing discoloration at both interfaces.

**Figure S7.** Cyclic voltammograms of the three-electrode cell using stainless steel as the working electrode and lithium as the counter and reference electrodes using TM14118 membrane at different temperatures, showing temperature-dependent interfacial reactions and polarization effects.

**Figure S8.** Photographs of TM14118 membrane in direct contact with lithium metal before (A) and after (B) storage at 60 °C for 48 h, showing no significant discoloration under direct thermal contact.

**Figure S9.** Cyclic voltammograms of the SN-free PEO–LiTFSI, PEO-free DEC‒SN‒LiTFSI, and both PEO- and SN-free DEC‒LiTFSI electrolytes, showing the contributions of the components to the oxidation process.

**Figure S10.** CV profiles of low-salt and low-SN-content membranes at different temperatures, showing SN as a major contributor to the oxidation peak.

**Figure S11.** (A, B) Impedance spectra of LTO and LCO electrodes paired with lithium before and after cycling with TM14118 at different temperatures, showing trends similar to those of LFP.

**Figure S12.** (A) CV profiles of SN-free LFP|PEO‒LiTFSI|Li cells at different temperatures and (B) their corresponding impedance before and after cycling.

**Figure S13.** Electrochemical impedance spectra of SS|TM14118|Li cells after cycling at different temperatures.

**Figure S14.** SEM image of electrospun P-NFM on TM14118-infused PVDF-HFP, showing interfacial layer formation resulting from P-NFM deposition.

**Figure S15.** EIS spectra of PVDF-HFP- and P-NFM-modified electrolytes and MLEM14118, showing negligible change in ionic conductivity at 30 °C.

**Figure S16.** Cyclic voltammograms of gel-MLEM14118 membrane, showing the reappearance of reduction and oxidation features upon expansion of the potential window.

**Figure S17.** EDX mapping of lithium after 100 cycles at 50 °C, showing the distribution of fluorine-containing species at the lithium interface.

**Figure S18.** FTIR spectra of pristine LFP and PEDOT-S-coated LFP, confirming successful polymer coating.

**Figure S19.** (A) CV profiles of SN-free PEDOT-S-LFP|PEO‒LiTFSI|Li cells at different temperatures and (B) their corresponding impedance before and after cycling.

**Figure S20.** Charge-discharge profiles showing differences in overpotential evolution during cycling for pristine and PEDOT-S-coated LFP paired with TM14118 and MLEM14118.

**Figure S21.** Cycling performance of pristine and PEDOT-S-coated LFP with SN-free PEO‒LiTFSI electrolyte, demonstrating the effectiveness of the PEDOT-S coating in stabilizing the cathode interface against reactions involving the PEO‒LiTFSI electrolyte.

**Figure S22.** Cycling performance of PEDOT-S-coated LFP with an active-material loading of ~5 mg cm^2^, showing the applicability of the dual-protection strategy to cathodes with increased active-material loading.

**Figure S23.** Charge-discharge profiles and cycling performance of pristine LCO and PEDOT-S-coated LCO paired with TM14118 and MLEM14118, respectively, demonstrating the applicability of the dual-protection strategy to higher-voltage cathodes.

**Figure S24.** N 1s XPS spectrum of the PVDF-HFP-protected stainless-steel interface after cycling with MLEM14118 between 2.5 and 4.3 V at 50 °C.

**Figure S25.** TEM-EDX mapping of pristine (A, C) and PEDOT-S-coated LFP (B, D) after cycling, showing the distribution of nitrogen-containing species across the particles and interfacial region.
